# Supplementary material for: Transcriptomic Insights into Mechanisms of Early Seed Maturation in the Garden Pea (Pisum sativum L.)
Source: Cells. 2020 Mar 23;9(3):779. doi: 10.3390/cells9030779 (PMC7140803; doi:10.3390/cells9030779)

Sprint−2,20(2)

Global Summary

%DE = 0.29

genes with fdr < 0.2 = 187 (78 + / 109 −)

genes with fdr < 0.1 = 167 (69 + / 98 −)

genes with fdr < 0.05 = 163 (69 + / 94 −)

genes with fdr < 0.01 = 146 (61 + / 85 −)

genes in genesets = 0

<FC> = 0

<t−score> = 0

<p−value> = 0

<fdr> = 0.71

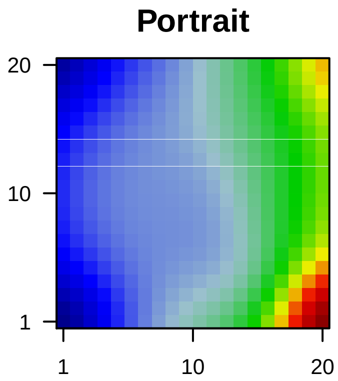

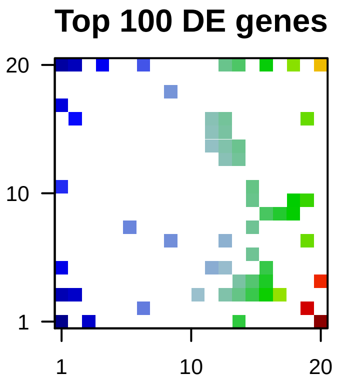

Global Genelist

Rank	ID	log(FC)	p−value	fdr	Metagene	Description
1	TRINITY_DN2	−0.17	2e−16	1e−15	14 x 3	
2	TRINITY_DN1	5.71	2e−16	1e−15	20 x 1	
3	TRINITY_DN1	0.11	2e−16	1e−15	12 x 15	
4	TRINITY_DN1	3.01	2e−16	1e−15	20 x 20	
5	TRINITY_DN5	−0.33	2e−16	1e−15	2 x 20	
6	TRINITY_DN5	0.13	2e−16	1e−15	15 x 11	
7	TRINITY_DN3	−0.11	2e−16	1e−15	1 x 17	
8	TRINITY_DN5	0.19	2e−16	1e−15	1 x 11	
9	TRINITY_DN5	−0.12	2e−16	1e−15	15 x 6	
10	TRINITY_DN2	2.73	2e−16	1e−15	20 x 1	
11	TRINITY_DN2	1.83	2e−16	1e−15	20 x 1	
12	TRINITY_DN3	−0.34	2e−16	1e−15	1 x 1	
13	TRINITY_DN3	0.31	2e−16	1e−15	19 x 10	
14	TRINITY_DN5	−0.38	2e−16	1e−15	1 x 1	
15	TRINITY_DN3	0.2	2e−16	1e−15	16 x 9	
16	TRINITY_DN3	0.1	2e−16	1e−15	15 x 10	
17	TRINITY_DN5	−0.14	2e−16	1e−15	14 x 4	
18	TRINITY_DN4	−0.51	2e−16	1e−15	15 x 3	
19	TRINITY_DN4	−0.83	2e−16	1e−15	1 x 1	
20	TRINITY_DN4	−0.47	2e−16	1e−15	18 x 20	

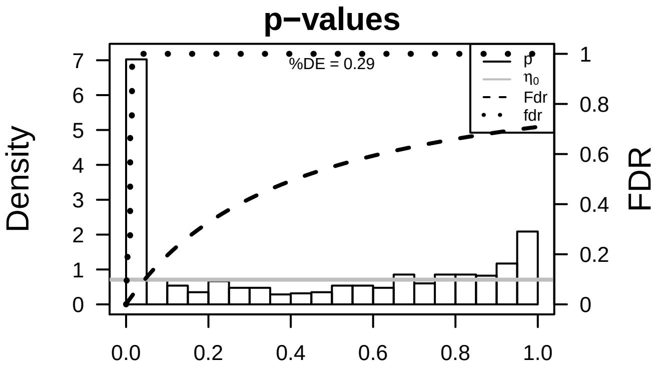

Supplement: Supplementary file 1 [file cells-09-00779-s001.zip › Supplementary materials/FileS3.zip!/FileS2/Hormones - Results/Summary Sheets - Samples/Sprint.2.20.2..pdf]
